# The natural history and evolution of dermatophytosis: Host immunity in acute and chronic infection

**DOI:** 10.1371/journal.ppat.1014264

**Published:** 2026-06-08

**Authors:** Aditya K. Gupta, Tong Wang, Anuradha Chowdhary, Ditte Marie L. Saunte, Roderick J. Hay, Vincent Piguet

**Affiliations:** 1 Division of Dermatology, Department of Medicine, Temerty Faculty of Medicine, University of Toronto, Toronto, Ontario, Canada; 2 Division of Dermatology, Women’s College Hospital, Toronto, Ontario, Canada; 3 Mediprobe Research Inc., London, Ontario, Canada; 4 Medical Mycology Unit, Department of Microbiology, Vallabhbhai Patel Chest Institute, University of Delhi, Delhi, India; 5 National Reference Laboratory for Antimicrobial Resistance in Fungal Pathogens, Vallabhbhai Patel Chest Institute, University of Delhi, Delhi, India; 6 Department of Clinical Medicine, Faculty of Health and Medical Sciences, University of Copenhagen, Copenhagen, Denmark; 7 PHOENIX Research Center, Department of Dermatology and Allergy, Copenhagen University Hospital - Herlev and Gentofte, Gentofte, Denmark; 8 St. John’s Institute of Dermatology, King’s College London, London, United Kingdom; University of Maryland, Baltimore, UNITED STATES OF AMERICA

## Etiopathogenesis of dermatophytosis—Historical understanding and current gaps

Research into dermatophytosis (tinea, ringworm) has been ongoing for nearly two centuries. From the initial descriptions of fungal structures in tinea capitis (scalp ringworm), to the cultivation and identification of an infectious etiology by David Gruby, and the pioneering work of Raymond Sabouraud in establishing dextrose agar, these foundational works have enabled the development of insights into the pathogenesis of superficial mycoses [1]. Ever since the global spread of *Trichophyton rubrum* coinciding with the end of World War II, dermatophytes have become the most common cause of fungal diseases in dermatological outpatient populations, typically manifesting on glabrous skin as erythematous, ring-shaped lesions with scaling, raised borders, and central clearing.

Although commonly considered a group of filamentous, keratinophilic fungi (*Trichophyton*, *Epidermophyton*, *Nannizzia*, *Paraphyton*, *Lophophyton*, *Microsporum*, *Arthroderma*, *Ctenomyces*, *Guarromyces*) with a predilection for the stratum corneum [2], dermatophytes can also cause dermal or subcutaneous infections in immunocompromised populations [3]; hence, the superficial localization of this infection is not strictly due to metabolic adaptations but also reflects host–pathogen interactions. Host immunity plays a critical role in controlling dermatophytosis, and, conversely, its dysregulation can lead to chronic infection and, in rare instances, invasive disease with a risk of systemic spread [3,4].

In recent years, clinicians and researchers have observed an epidemiological shift within the zoophilic *Trichophyton mentagrophytes* complex, characterized by the emergence of anthropophilic lineages with adaptations to human hosts. This includes *T. mentagrophytes* ITS genotype VII, which has been associated with sexual transmission [5], and *T. indotineae* (*T. mentagrophytes* ITS genotype VIII), which has acquired antifungal resistance, particularly to terbinafine [6].

Since its initial reporting around 2015 in the Indian subcontinent, *T. indotineae* has emerged as a cause of unusually widespread and treatment-resistant dermatophytoses and has now spread intercontinentally, with rising case numbers reported in the United States [7], Canada [8], and Europe [9,10]. Its global dissemination follows a largely clonal evolutionary pattern, driven by single-nucleotide variations (SNVs) in the squalene epoxidase gene that confer decreased terbinafine susceptibility [11,12]. Concurrent with its rise, clinical features once considered characteristic of dermatophytosis have evolved, notably widespread lesions on the trunk (tinea corporis) and groin (tinea cruris) that lack central clearing, with some exhibiting hyperpigmentation and variable degrees of inflammation (minimal to erythrodermic) [6,13]. *T. indotineae* patients often experience a chronic infection course with recurrent episodes, and household transmission is common.

**Fig 1:**
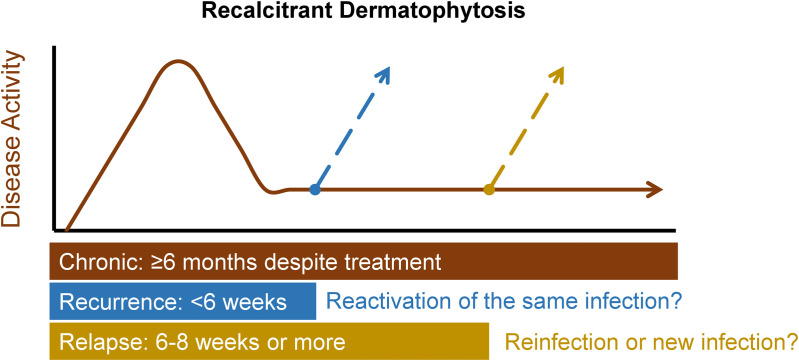
Proposed clinical definitions of chronic, recurrent, and relapsing dermatophytosis in the current outbreak situation due to *Trichophyton indotineae* [14,15].

In an effort to better categorize disease progression amidst recent outbreaks of *T. indotineae* infections, expert working groups have adopted the term “recalcitrant dermatophytosis” as an umbrella term for three commonly encountered clinical variants: chronic, recurrent, and relapse [14,15] (Fig 1). Based on clinical observations, patients with persistent disease for 6 months or more, with or without recurrent episodes, are considered chronic. Recurrent dermatophytosis refers to the reoccurrence of lesions within less than 6 weeks of treatment completion, while relapse refers to the reoccurrence of lesions after a longer period of 6–8 weeks.

In the absence of mycological and immunological evaluations, interpreting these clinical categories can be challenging, as lesion recurrence may signal either exogenous reinfection or endogenous reactivation. Further compounding this terminological confusion, in the tuberculosis literature, a recurrence refers to a new disease episode after treatment success, which is differentiated by molecular testing into either reinfection by a new strain or relapse of the previous strain [16].

In dermatophytosis, regardless of the underlying cause of lesion recurrence, studies have shown that immunocompetent individuals typically experience an acute course of infection and are more resistant to reinfection, with milder symptoms [17–19]. Hence, immunological profiling should be subject to further clinical investigation, as this approach may offer greater predictive value for clinical outcomes. Given the multitude of host-, pathogen-, and treatment-related factors that influence the course of infection, we herein seek to re-examine the natural history and evolution of dermatophytosis focusing on host resistance mechanisms. New insights into the role of sensory neurons in skin immunity are also discussed.

## Natural history of dermatophytosis—Insights from systematic longitudinal observations and the “classical” view of dermatophyte immunity

Managing new and ongoing outbreak scenarios can be improved by a better understanding of their natural history [20]. The natural history of an infectious disease—a reflection of host–pathogen interactions—generally refers to the period from exposure and infection through symptom onset to eventual outcome, in the absence of external intervention [20]. However, outside of historical records or animal model experiments, it would be unethical to observe the natural history of an infectious disease outbreak without providing medical care; hence, a modified “host-pathogen-care” framework has been proposed [20].

**Fig 2:**
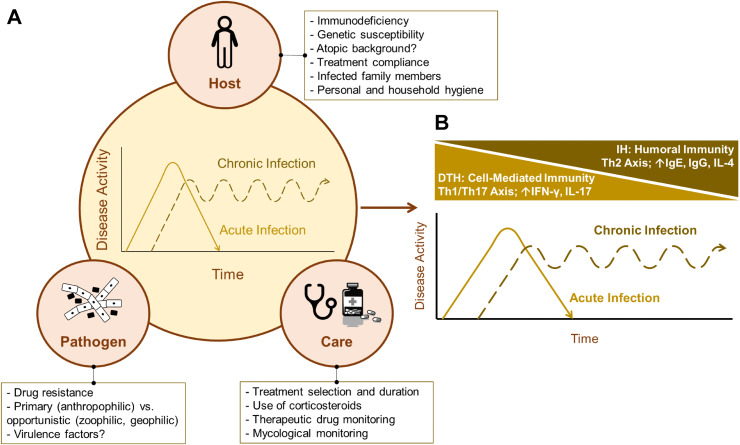
The natural history of dermatophytosis. (A)A proposed host-pathogen-care framework for studying the natural history of dermatophytosis, which may follow either an acute course or a chronic course. While pathogen- and care-related factors contributing to chronicity have been studied, less is known about host determinants. Host determinants include immunodeficiencies; notably, misuse of corticosteroid fixed-dose combinations (e.g., clobetasol propionate admixed with terbinafine) has been associated with the emergence of *Trichophyton indotineae*. Inborn errors of immunity, such as CARD9 deficiency, are also linked to chronic infection and can increase the risk of invasive disease (deep dermatophytosis). Atopy is associated with chronic dermatophytosis due to overlap in humoral immune pathways. Pathogen-related factors include antifungal resistance, particularly terbinafine resistance in light of *T. indotineae* outbreaks. Zoophilic and geophilic dermatophytes tend to cause more inflammatory, acute lesions, whereas anthropophilic dermatophytes are associated with chronic infections with less inflammation. Less is known about dermatophyte virulence factors (e.g., secreted enzymes, toxins, pH-responsive transcription factors) that can influence the course of infection. For care-related factors, appropriate treatment selection informed by antifungal susceptibility testing and adequate treatment duration guided by mycological monitoring—under the principle of antifungal stewardship—may reduce the risk of chronic infections. Therapeutic drug monitoring is recommended for triazoles (e.g., itraconazole, voriconazole, posaconazole) to prevent sub- or supratherapeutic levels. (B) “Classical” view of cell-mediated immunity and humoral immunity in acute and chronic dermatophytosis. Patients with acute dermatophytosis exhibit protective cell-mediated immunity, which manifests as a delayed-type hypersensitivity reaction upon exposure to Trichophytin. This response is classically thought to involve infiltrating CD4^+^ T-helper 1 (Th1) and Th17 cells from skin-draining lymph nodes, resulting in the production of IFN-γ and IL-17. By contrast, a non-protective Th2-mediated humoral responsive (IgE, IgG, IL-4) is associated with chronic dermatophytosis, which manifest as immediate-type hypersensitivity to Trichophytin. DTH, delayed-type hypersensitivity; IH, immediate-type hypersensitivity. Two of the visual elements shown were reused in accordance with the Creative Commons Zero 1.0 Public Domain License.

In dermatophytosis, a degree of clinical heterogeneity has long been recognized ever since the global spread of *T. rubrum* more than 70 years ago [18,21], with some patients undergoing an acute, sometimes self-limited course of infection, while others develop chronic, recalcitrant infections marked by dissemination across multiple anatomical sites and variable, atypical clinical morphologies (Fig 2A). This warrants a systematic, longitudinal observational approach to assess underlying risk factors and to re-direct the natural history through targeted interventions. The following sections focus primarily on conventional and newer insights into host immunity. Readers are referred to recent reviews detailing pathogen- and care-related factors contributing to chronicity [6,22,23].

Differing skin hypersensitivity reactions to antigen preparations from *Trichophyton* (“Trichophytin”)—either immediate-type hypersensitivity (IH) or delayed-type hypersensitivity (DTH)—have been suggested as hallmarks of the natural history of dermatophytosis (Fig 2B) [24]. These preparations contain fungal, allergenic glycoproteins composed mainly of D-galactose and D-mannose, with approximately three short peptide chains [25]. Secreted keratinases by dermatophytes could also elicit similar reactions [26]. In a study of human volunteers who were not previously sensitized to Trichophytin, experimental exposure to dermatophytes resulted in the induction of IH and/or DTH reactions to Trichophytin [27].

In individuals with a history of acute infection who exhibited only DTH, Jones et al. demonstrated that experimental re-infection of *T. mentagrophytes* resulted in rapid disease clearance without dissemination to other skin surfaces, even in the absence of treatment, with some showing no visible disease at lower inoculum levels [17]. In contrast, experimental infection of chronic dermatophytosis patients induced disseminated, coalescing lesions and delayed healing—particularly in those exhibiting IH alone compared with those displaying both IH and DTH or DTH alone—and was associated with a lesser degree of inflammation. Interestingly, chronically infected patients with a mix of IH and DTH were considered to represent an “intermittent” immunological category, where infections were more inflammatory but less extensive and persistent than those observed in chronic patients with IH alone [17,18]. A longitudinal observation of an atopic patient who developed chronic dermatophytosis revealed an initial DTH response, followed by a turnover to the IH response and increase in serum IgE, coinciding with reduced inflammation and the development of a disseminated infection at other body sites [28].

DTH reflects cell-mediated immunity, conventionally thought to involve T helper 1 (Th1) and Th17 responses, and is associated with acute infection and zoophilic dermatophytes (e.g., *T. mentagrophytes* var. *mentagrophytes*) [24,29]. This response is marked by elevated interferon-gamma (IFN-γ) and interleukin-17 (IL-17), lower titers of dermatophyte-specific IgG and undetectable IgE, with skin reactions (erythema, edema, induration) peaking at 24–48 hours post-injection [18]. Insights from animal models of acute dermatophytosis are summarized in Box 1.

**Box 1. Acute dermatophytosis in animal models**

Using mouse models of acute dermatophytosis, a primary infection is characterized by skin inflammation, erythema, and crust formation, which progress and peak around the first week post-infection [19,30]. Histopathologic findings include an increase in epidermal thickness and prominent neutrophil infiltration surrounding the mycelium, with lymphocytes and macrophages appearing later during infection [19,30]. In particular, skin crusts contain neutrophils surrounding the mycelium and eventually develop into a zone of inflammatory cells that becomes separated from the epidermis and is subsequently shed [19]. Immunity can be conferred on mice by adoptive transfer of T cells from immune donor animals [31].

In skin-draining lymph nodes of acutely infected mice, Heinen and colleagues demonstrated that CD4^+^ T cells polarize toward the Th1/Th17 axis, producing IFN-γ and IL-17A, which synergistically attenuate disease progression [30]. IFN-γ is thought to modulate the fibrinolytic system, thereby facilitating macrophages and neutrophils to access and eliminate fungi, as well as upregulate the phagocytic activity of macrophages by promoting M1 polarization. IL-17A, either alone or together with IL-22, promotes the differentiation and chemotaxis of neutrophils as well as stimulates keratinocytes to produce antimicrobial peptides. Following acute resolution, reinfection is associated with reduced clinical severity, a more rapid and pronounced increase in epidermal proliferation, and faster clearance of fungal elements with infiltration of neutrophils, macrophages, and lymphocytes, as well as increased Th1/Th17 responses [19,30].

IH reflects humoral immunity and a Th2 type response, often associated with chronic infection and anthropophilic dermatophytes (e.g., *T. rubrum*, *T. indotineae*) [24,29,32]. This response is marked by elevated immunoglobulin E (IgE), dermatophyte-specific IgG, and IL-4, with a wheal and flare skin reaction occurring at 5–20 min after injection. In this setting, binding of dermatophyte antigens to IgE triggers mast cell degranulation, releasing histamine; this pathway may be positively regulated by the production of IL-4 by Th2 cells that mediates B-cell class switching to IgE [24]. Early studies have identified the IH response in association with reduced leukocyte migration and concomitant atopic conditions, including chronic infections caused by *T. rubrum* and *T. concentricum* [33,34]. Insights from animal models of chronic dermatophytosis are summarized in Box 2.

**Box 2. Chronic dermatophytosis in animal models**

In animal models, although dermatophyte infections typically follow an acute course, the lack of functional T-cell immunity (athymic mice) and steroid-induced immunosuppression can divert the course of infection from acute to chronic, characterized by prolonged infection, enlarged lesions with new satellite lesions, and reduced inflammation [35,36]. Through intraperitoneal injection of adjuvant and dermatophyte antigens, Hay and colleagues established a chronically infected mouse model that showed an increased number of mast cells compared to acutely infected mice, some which have undergone degranulation, possibly leading to elevated histamine levels [37].

Chronic dermatophytosis sits at an interesting intersection with atopic diseases [24]. In addition to skin sensitization associated with urticaria [38], it has been hypothesized that *Trichophyton* antigens may sensitize the upper airways, based on the observed association between bronchial asthma or allergic rhinitis and chronic dermatophytosis [21,28,39]. In a study of asthmatic individuals with concomitant dermatophytosis and an IH skin response, almost all patients showed hypersensitivity reactions to bronchial and nasal challenges with *Trichophyton* antigens, compared to none in healthy controls and in asthmatic individuals without dermatophytosis [40]. In these patients, elevated IgE may not only impair cell-mediated immunity against dermatophytes [41], but also potentially exacerbates concomitant atopic diseases. Notably, antifungal treatments can alleviate atopic symptoms [39,40].

Mechanisms underlying this dichotomous DTH and IH response are unclear and likely involve both host and pathogenicity factors. It is generally assumed that the ecological niche of dermatophytes influences the course of infection, whereby zoophilic dermatophytes tend to cause more acute, inflammatory infections, reflecting opportunism and lack of adaptations to human hosts, whereas anthropophilic dermatophytes evolve to cause mild, chronic infections, resulting in higher transmissibility from person to person. Accordingly, the DTH response may be more frequently observed with zoophilic *T. mentagrophytes* var. *mentagrophytes* and the IH response with anthropophilic *T. rubrum* [21,42]. In the case of *T. indotineae*, its more recent ecological adaptation to humans may partially explain the variable degrees of inflammation.

Other proposed risk factors for developing chronicity include the location of infection. In tinea pedis, the “moccasin” variety—characterized by dry, scaly lesions of the plantar, medial, or lateral aspects of the foot that are prone to fissures—is often associated with chronic infection, possibly due to reduced epidermal integrity [17,18]. This condition also increases the risk of secondary bacterial infections and the possible hematogenous spread of dermatophyte antigens, which can trigger a hypersensitivity reaction in distant anatomical sites, known as an “id” reaction (dermatophytid or trichophytid) [29,43]. Similarly, epidermal barrier defects due to palmoplantar keratoderma or lamellar ichthyosis are also associated with chronic dermatophytosis [44].

Antifungal resistance, by causing protracted antigen exposure, may lead to anergy of cell-mediated immunity [24]. This risk factor is receiving increased attention due to the evolution of *T. indotineae* and terbinafine resistance. Although dermatophyte resistance to griseofulvin and fluconazole has been demonstrated previously, recent developments in terbinafine resistance are particularly concerning due to its fungicidal activity and favorable safety profile, which have made it the preferred first-line treatment [22]. The next available treatment option, itraconazole, may also see waning efficacy due to recent microbiological evidence of resistance, corroborated by the detection of *ERG11B/CYP51B* overexpression in *T. indotineae* isolates [8]. A rising incidence of antifungal resistance is also seen with other dermatophytes, such as *T. rubrum*.

## Current-day scenario—*Trichophyton indotineae* and the role of local and systemic immunosuppression

Localized immunosuppression due to the misuse of steroid-antifungal ointments is also considered a major factor in the current outbreaks of *T. indotineae* across the Indian subcontinent. These products often contain either clobetasol propionate (ultrapotent) or betamethasone dipropionate (high potency) [45]. Skin treated with these potent steroids shows suppression of Langerhans cells (LCs) [46], which are skin-resident dendritic cells typically abundant in the epidermis that express major histocompatibility complex class II, and function as antigen-presenting cells during dermatophyte infection. Accordingly, Bhat and colleagues recently demonstrated that perilesional samples from Indian patients with chronic dermatophytosis showed a reduced number of CD1a^+^ LCs, compared with patients who recovered from acute dermatophytosis [47]. Similarly, a reduced number of CD1a^+^ LCs was also found in patients with disseminated dermatophytosis affecting three or more anatomical sites, compared with healthy controls [48]. Patel and colleagues further reaffirmed the risk of corticosteroid use, showing that exposure to topical corticosteroids as first-line treatment, along with cumulative exposure, was significantly associated with chronic infections, characterized by disease durations of more than one year with >10% body surface area (BSA) affected [49]. This leads to skin atrophy (striae and hypopigmentation), severe pruritus, and steroid-modified lesions that resemble eczema.

Furthermore, the high burden of prediabetes and type 2 diabetes mellitus may compound the immunosuppressive effects of topical corticosteroids and further exacerbate the burden of dermatophytosis across the Indian subcontinent. In a cohort study, diabetic patients (HbA1C ≥ 6.5%) with dermatophytosis were more likely to develop chronicity and recurrence, with a higher percentage of BSA affected, than non-diabetic patients with dermatophytosis [50]. For every point increase in HbA1c, an incremental increase in the risk of dermatophytosis was also reported, possibly due to systemic immunosuppression or epidermal barrier dysfunction secondary to neuropathic ulcers [51]. This risk was particularly higher among prediabetic patients than among diabetic patients, which may be due to fewer medical interventions or less proactive footcare.

The culmination of unregulated steroid use and diabetes can lead to systemic T-cell dysregulation, resulting in higher risks of chronic dermatophytosis. Jha et al. demonstrated a reduced number of circulating Th1 (CD4^+^IFN-γ^+^) and Th17 (CD4^+^IL-17^+^) cells, along with an increased number of circulating Th2 (CD3^+^IL-4^+^) cells, in recurrent dermatophytosis patients—mainly due to infection by the *T. mentagrophytes* complex (*T. indotineae*)—compared to both acute dermatophytosis patients and healthy controls [52]. This skewed Th2-type response was corroborated by elevated serum IgE and the absence of a DTH response [52]. Serum IgE levels also showed a significant positive correlation with both the percentage of BSA affected and disease duration in chronically infected patients [53]. Additionally, Rai and colleagues reported upregulation of circulating regulatory T cells (Treg; CD4^+^CD25^+^FoxP3^+^) along with paradoxical activation of a Th17 population (CD4^+^CD161^+^IL23R^+^) [54]. Both Treg and Th17 populations expanded upon exposure to Trichophytin and were detected at higher levels compared to healthy controls. This is postulated to be the result of cell plasticity and trans-differentiation between Treg and Th17 cells, which may also contribute to the development of chronicity [54]. It should be noted that these immunological findings in *T. indotineae* infections are similar to those reported previously in persistent *T. rubrum* infections, irrespective of steroid use.

Lastly, the introduction of targeted immunotherapies, which have revolutionized the management of autoimmune diseases, cancers, and solid organ or stem cell transplantation, may increase the risk of infectious adverse events and warrants further study. In particular, the use of IL-17 inhibitors (e.g., bimekizumab, secukinumab) in psoriasis patients is associated with a significantly increased risk of dermatophytosis [55]. In two case reports, the use of pembrolizumab (a PD-1 inhibitor) and infliximab (a TNF-α inhibitor) were attributed to the development of deep infections in patients with pre-existing dermatophytosis [56,57].

## Inborn errors of immunity (primary immunodeficiency)

Besides acquired immunodeficiencies, inborn errors of immunity are also being recognized as factors contributing to fungal infections, which could predispose vulnerable populations to chronic dermatophytosis. CARD9 (caspase recruitment domain-containing protein 9) is an adaptor molecule primarily expressed in myeloid cells, particularly LCs [58]. It functions downstream of C-type lectin receptors (Dectin-1, Dectin-2), which are pattern recognition receptors for dermatophyte antigens, and forms a filamentous complex with BCL10 and MALT1 upon receptor activation in dendritic cells. In a *T. rubrum*-infected mouse model with CARD9-deficient dendritic cells carrying the R101C missense mutation, which leaves CARD9 in an autoinhibited state, the interaction between BCL10 and CARD9, as well as the downstream activation of NFĸB (nuclear factor-ĸB), were impaired [58]. This CARD9 deficiency led to downregulated cytokine and chemokine responses, a prolonged infection course with an increased fungal load, decreased infiltration of neutrophils and monocytes, and downregulation of Th17 cells [58].

Concerning macrophages, experiments modeling other fungal infections have demonstrated that CARD9 promotes polarization toward the M1 phenotype, whereas CARD9 deficiency skews macrophages toward the M2 phenotype, possibly through upregulation of TREM2 (triggering receptor expression on myeloid cells 2) [59,60]. Enrichment of TREM2^hi^ macrophages has also been associated with accumulation of Tregs and loss-of-function or exhaustion-like Th1 cells [60]. Clinically, *CARD9* mutations are significantly associated with the development of deep, invasive dermatophytosis [3].

Even in individuals without clinically recognized immunodeficiency states, genome-wide association and exosome sequencing studies have identified markers associated with dermatophytosis. Using the UK Biobank, SNVs in *TINAG* and *KLK3* were significantly associated with the clinical diagnosis of dermatophytosis, which may impair immunity and epidermal integrity, requiring further functional validation [61,62]. In Israeli patients with chronic dermatophytosis, *CARD9* and *FOXN1* mutations may also contribute to multidrug resistance, in addition to microbiological factors [63].

Semaphorins, a group of secreted glycoproteins, act as ligands for several cellular pathways, including axon development, and exhibit immunomodulatory functions [64]. In inflammatory conditions, the transmembrane semaphorin 6A (SEM6A) is preferentially expressed in LCs and may mediate IFN-γ-dependent activation and migration to regional lymph nodes [64]. In a genomic investigation of children with chronic tinea capitis caused by *T. tonsurans*, variations of the *SEM6A* gene were identified as a predisposing factor, potentially pointing by a defective activation ability of LCs [65].

Another hint at a dysregulated innate immunity in chronic dermatophytosis is natural killer (NK) cells. Although not typically considered skin-resident lymphocytes, NK cells play an important role in immune surveillance through the recognition of self-proteins and non-self-proteins, mediating both direct and indirect elimination of infected or neoplastic cells [66]. In a study of household transmissions of dermatophytosis, siblings with chronic tinea corporis exhibited a more activated circulating NK cell population (higher expression of NKG2D and lower expression of NKG2A) with an increased expression of chemokine receptors (CXCR3, CXCR4)—potentially reflecting differences in skin-homing—compared to noninfected siblings [67].

Recent research has recognized NK cells for their skin distribution, either during fetal development or during skin pathological changes whereby circulating NK cells are recruited [66]; in particular, a subset of skin-resident NK cells—marked by CD56^bright^, Tcf7^hi^, and CD69^hi^—may readily respond to infections by secreting cytokines (e.g., IFN-γ), which in turn could upregulate Th1/Th17 responses [68]. Additionally, there are seemingly important interactions between NK cells and LCs. In *Candida albicans* infection, LCs attenuated skin inflammation by tissue-resident NK cells (CXCR6^+^CD49a^+^), suggesting an immunoregulatory function [69]. In light of recent findings of tissue-resident lymphoid cells in the skin, which has led to the proposal of the skin as a secondary lymphoid organ, further research is needed to decipher their roles in dermatophytosis [66].

## Conclusions and future outlook

Although dermatophytosis is commonly regarded as a mild infection, this fungal disease appears to be evolving due to new human pathogens emerging from the zoophilic *T. mentagrophytes* complex. As with the historical spread of *T. rubrum*, the emergence of *T. indotineae* is causing a paradigm shift in management. In addition to antifungal resistance contributing to the spread of *T. indotineae*, there are open questions about its atypical clinical presentations, the development of chronicity, recurrences, and reinfections, and the underlying role of primary or acquired immunodeficiencies. The current body of research suggests that the development of chronic dermatophytosis is multifactorial, requiring clinical, mycological, and immunological evaluations. Establishing a reproducible animal model can advance our understanding; however, anthropophilic dermatophytes (*T. rubrum*, *T. indotineae*) do not naturally infect mice, and guinea pig models—which are naturally infected by *T. benhamiae*—are complicated by high costs and the lack of immunological assays. Recent efforts to optimize a mouse model can help uncover potential neuroimmune interactions in dermatophytosis (Box 3) [70,71]. Furthermore, establishment of monocyte cell cultures or mouse models with targeted alterations, such as CARD9 deficiency, may also elucidate the pathogenesis of deep dermatophytosis [58,59].

**Box 3. New perspectives from other fungal skin diseases—Neuroimmune interactions and skin-resident lymphoid cells**

**Fig 3:**
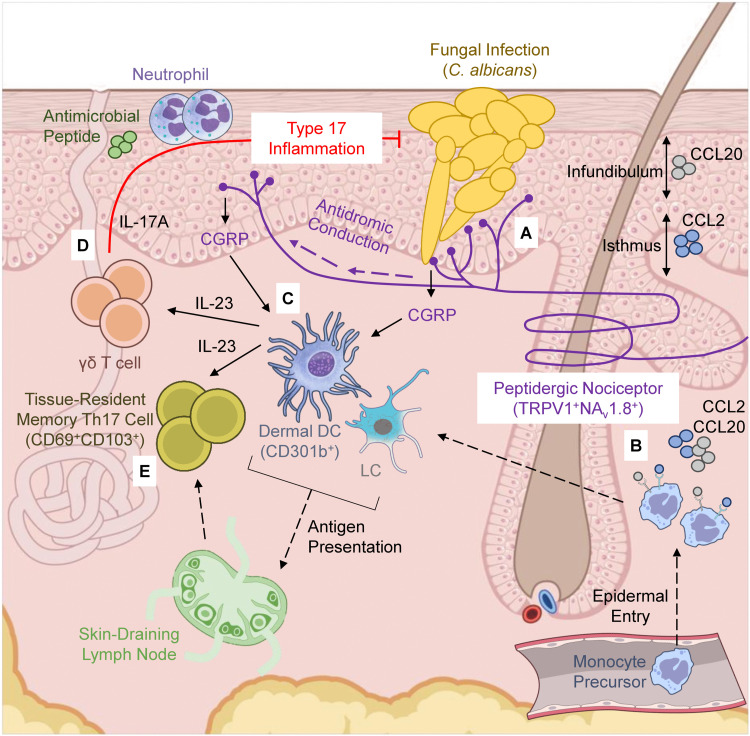
Proposed neuroimmune interactions in the follicular microenvironment during fungal infections. **(A)** Peptidergic nociceptors (TRPV1^+^NA_v_1.8^+^) detect fungal pathogens and initiate action potentials, triggering neighboring nerve endings via an antidromic reflex (seconds), followed by the release of neuropeptides (e.g., CGRP) into the hair follicle (minutes). **(B)** Upon sensing inflammatory signals, keratinocytes localized to the hair follicle recruit monocyte precursors through the release of chemokines CCL2 and CCL20 (hours). **(C)** Following differentiation of these monocyte precursors, activation of CD301b^+^ dermal dendritic cells (DCs) or closely related Langerhans cells (LCs) by CGRP induces the release of IL-23, which in turn promotes **(D)** type 17 inflammation by skin-resident lymphoid cells (e.g., γδ T cells), thereby executing an antifungal response (days). **(E)** Following resolution of infection, IL-23 also helps maintain tissue-resident memory T-helper (Th) 17 cells, which are primed by dermal DC populations presenting fungal antigens and mediate protection against reinfection. Nine of the visual elements shown were reused under the Public Domain or in accordance with the Creative Commons Zero 1.0 Public Domain License.

The skin contains a network of sensory neurons that coordinate with the local immune system to mediate inflammation and early antimicrobial responses [72], with the follicular microenvironment serving as a key hub that is responsive to neuropeptides and recruits monocyte precursors [73] (Fig 3). The hair follicle is innerved by peptidergic nociceptors [74], which release neuropeptides such as CGRP and substance P that can trigger pain sensation and are also implicated in innate immunity [72]. Upon pathogen recognition—such as directly through Dectin-1 or indirectly through ATP activation—neuropeptide release can initiate an immune cascade, including IL-17/IL-22-mediated responses against fungal invaders [72]. This process is further facilitated by the recruitment of monocyte precursors (Gr1^hi^) at the hair follicle through the localized expression of CCL2 (isthmus) and CCL20 (infundibulum), corresponding to the chemokine receptors CCR2 and CCR6, respectively [73].

In psoriasiform inflammation, peripheral nociceptive neurons (TRPV1^+^NA_v_1.8^+^) stimulate dermal dendritic cells to produce IL-23, which in turn activates gamma delta (γδ) T cells and initiates the IL-17/IL-22 inflammatory cascade [75]. A similar pathway has been demonstrated in cutaneous candidiasis by Kaplan and colleagues, in which TRPV1^+^ neurons detect *C. albicans* and release the neuropeptide CGRP, thereby stimulating dermal dendritic cells (CD301b^+^) to produce IL-23 [76]. This leads to the proliferation of γδ T cells and their production of IL-17A, resulting in a reduction in fungal burden. Furthermore, following resolution of infection, IL-23 production can help reduce the risk of reinfection by maintaining tissue-resident memory Th17 cells (CD69^+^CD103^+^) [77].

A notable feature of neuroimmune interactions is its rapid responsiveness. Through antidromic conduction, action potential—mediated by ion channels such as NA_v_1.8—downstream of receptor activation can stimulate neighboring nerve endings, amplifying neuropeptide release and enabling rapid, localized activation of immune cells, including at uninfected skin sites (i.e., “anticipatory immunity”) [78].

The role of γδ T cells in dermatophytosis remains unclear, as studies indicate that cell-mediated immunity is characterized by circulating and infiltrating Th1 and Th17 cells, associated with IFN-γ and IL-17A, respectively [4,30,52]. However, innate sources of IL-17 have been demonstrated in zoophilic *Microsporum canis* and geophilic *Nannizia gypsea* infections [70]. Using a mouse model, two populations of activated/memory IL-17A^+^ T cells—γδTCR^int^ and CD8/CD4 double-negative βTCR^+^—were identified. These skin-resident populations coordinated antifungal responses together with ILC3 (type 3 innate lymphoid cells), without involving CD4^+^ T cells recruited from regional lymph nodes [70]. These findings highlight an enrichment of innate-like effector cells in skin immunity, which respond rapidly to fungal pathogens, possibly via interactions with nociceptive neurons. Dysregulation of this pathway may contribute to the development of chronic dermatophytosis, as neuroimmune interactions can also skew towards a Th2-type response in autoimmune diseases and atopy, warranting further research [72].

The extent to which dermatophyte virulence factors affect host immunity also remains under-investigated. Previous studies have shown that cytoplasmic and exoantigens preparations from *T. rubrum* may modulate T-cell immunity in chronic dermatophytosis patients [79], and that melanogenesis may confer increased resistance to phagocytic killing [80]. In *T. indotineae*, hemolytic activity has recently been reported, which may help explain disseminated skin infections and hyperpigmentation [81]. These features of dermatophytic fungi need to be explored further.

Using experimental infection models, recent investigations have identified serine proteases, particularly the subtilisins, as markers of dermatophyte infection in both anthropophilic (*T. rubrum*) and zoophilic (*T. benhamiae*) species [82]. Interestingly, subtilisin 6 isolated from *T. rubrum* was previously investigated as an antigen (Tri r 2) and was found to induce proliferation of peripheral blood mononuclear cell (PBMC) cultures from both DTH and IH responders [83,84]. Furthermore, a 20-mer immunodominant epitope (YIIDTGIDIDHEDFQGRAKW) induced markedly higher proliferative response in DTH responders than IH responders [83], as well as higher levels of IL-5 and IL-10, while IFN-γ levels remained paradoxically comparable [85]. In healthy individuals, PBMC cultures stimulated with a 10-mer Tri r 2 epitope (YIIDTGIDID) exhibited a higher proliferative response and elevated IFN-γ levels than those from patients with extensive dermatophytosis [86]. As has been demonstrated successfully for *T. verrucosum* [87], further genomic investigations of the subtilisin family may facilitate the development of prophylactic and therapeutic vaccination strategies against *Trichophyton* spp.

With the introduction of targeted immunotherapies, it may be possible to improve the management of chronic dermatophytosis when combined with effective antifungals. Granulocyte-macrophage colony-stimulating factor (GM-CSF) regulates a wide range of myeloid cells, including the neutrophil response, and its administration may shorten the recovery time of febrile neutropenic patients with invasive fungal diseases (IFDs) [88]. Although studies have reported the efficacy of adjuvant GM-CSF therapy following the development of IFDs, high-quality evidence remains scarce [89–92]. In an immunocompromised patient with chronic mucocutaneous *C. albicans* infection accompanied by elevated serum IgE and IL-4 levels, administration of GM-CSF led to complete clearance of skin and mucosal lesions and partial clearance of nail lesions, with sustained improvement observed after 2 years [93].

In dermatophytosis, the therapeutic potential of GM-CSF is demonstrated by its upregulation in keratinocytes upon *T. rubrum* infection and its downregulation in chronically infected patients [94,95]. Similarly, IFN-γ is also downregulated in chronic dermatophytosis patients [52], which altogether suggests that this population could be a candidate for adjuvant immunotherapies that improve the efficacy of conventional antifungals while reducing treatment duration and associated side-effects. Further research is warranted develop innovative treatment strategies in response to the changing epidemiology of dermatophytosis.
